# Growth Hormone-Releasing Hormone and Its Analogues: Significance for MSCs-Mediated Angiogenesis

**DOI:** 10.1155/2016/8737589

**Published:** 2016-09-27

**Authors:** Xiangyang Xia, Quanwei Tao, Qunchao Ma, Huiqiang Chen, Jian'an Wang, Hong Yu

**Affiliations:** ^1^Department of Ultrasound, The Second Affiliated Hospital of Zhejiang University School of Medicine, Hangzhou 310009, China; ^2^Hangzhou Leading Pharmatech Co., Ltd., Hangzhou 311100, China; ^3^Department of Cardiology, The Second Affiliated Hospital of Zhejiang University School of Medicine, Hangzhou 310009, China; ^4^Cardiovascular Key Laboratory of Zhejiang Province, Hangzhou 310009, China; ^5^Department of Cardiology, The Second Hospital of Shandong University, Jinan, Shandong 250033, China

## Abstract

Mesenchymal stromal cells (MSCs) are promising candidates for regenerative medicine because of their multipotency, immune-privilege, and paracrine properties including the potential to promote angiogenesis. Accumulating evidence suggests that the inherent properties of cytoprotection and tissue repair by native MSCs can be enhanced by various preconditioning stimuli implemented prior to cell transplantation. Growth hormone-releasing hormone (GHRH), a stimulator in extrahypothalamus systems including tumors, has attracted great attentions in recent years because GHRH and its agonists could promote angiogenesis in various tissues. GHRH and its agonists are proangiogenic in responsive tissues including tumors, and GHRH antagonists have been tested as antitumor agents through their ability to suppress angiogenesis and cell growth. GHRH-R is expressed by MSCs and evolving work from our laboratory indicates that treatment of MSCs with GHRH agonists prior to cell transplantation markedly enhanced the angiogenic potential and tissue reparative properties of MSCs through a STAT3 signaling pathway. In this review we summarized the possible effects of GHRH analogues on cell growth and development, as well as on the proangiogenic properties of MSCs. We also discussed the relationship between GHRH analogues and MSC-mediated angiogenesis. The analyses provide new insights into molecular pathways of MSCs-based therapies and their augmentation by GHRH analogues.

## 1. Introduction

Growth hormone (GH), secreted by the somatotropes in the anterior part of pituitary gland, is the predominant hormone that regulates linear growth. Its production and secretion are controlled by growth hormone-releasing hormone (GHRH), along with the somatostatin, GH itself, and downstream factors such as insulin growth factor 1 (IGF-1). GHRH and its receptors are expressed not only in the hypothalamus and pituitary but also in peripheral tissues. Thus, in addition to modulating GH release, GHRH indirectly regulates the proliferation of cells in multiple other tissues including tumor cells through a GHRH/GH/IGF-1 axis. GHRH can also directly regulate cell growth through paracrine/endocrine mechanisms by binding to the GHRH receptor on target cells. Because of this, synthetic agonists and antagonists of GHRH have attracted wide attention in recent years as global regulators of cell growth with therapeutic potential including tissue regeneration and tumor suppression, respectively. GHRH has been shown to stimulate angiogenesis in human neuroendocrine tumors by promoting VEGF secretion [1]. Agonists of GHRH applied to the post infarct myocardium improved cardiac remodeling and helped resolve ischemia [2]. GHRH antagonists have been widely used to inhibit angiogenesis and proliferation of tumor cells in prostate cancer [3], endometrial cancer [4], non-small cell lung cancer [5], and ovarian cancer [6].

Mesenchymal stromal cells (MSCs), produced in the bone marrow as well as peripheral tissues, are recognized by their plastic adherence, expression of a panel of specific cell surface markers, and multipotent differentiation potential. In part because of their multipotency and immune-privilege properties, MSCs have been widely used to promote tissue regeneration including reconstruction of blood vessels [7, 8], cardiac repair [9], and angiogenesis [10–12]. However, the full regenerative potential of MSCs for clinical application is limited by poor posttransplantation engraftment and survival of native MSCs in the adverse microenvironment of a myocardial infarct of other ischemic circumstance [13]. Various interventions have been used with some success to enhance MSC survival including genetic modification [14], hypoxia preconditioning [15, 16], and pretreatment with chemical agents such as erythropoietin and unsaturated fatty acids [17, 18]. Work from our laboratory and others confirms that GHRH and its analogues can enhance angiogenesis in the infarcted heart and markedly enhance the regenerative properties of MSCs [19, 20]. Other laboratories have also clearly shown the converse that GHRH antagonists powerfully inhibit angiogenesis and growth of lung cancer cells [21], prostate cancer cells [22], glioblastomas cells [23], and breast cancer cells [24]. Therefore, we speculate that GHRH is a natural modulator of MSC activity, and agonists or analogues of GHRH may be the key to optimizing the regenerative properties of these cells for cardiovascular indications.

Here, we summarize current knowledge on the effects of GHRH analogues on normal and malignant cells and the potential application of GHRH analogues to optimize the proangiogenic and reparative properties of MSCs.

## 2. GHRH and Its Analogues

### 2.1. The GHRH/GH/IGF-1 Axis

The GHRH/GH/IGF-1 axis is a fundamental endocrine regulatory pathway that contributes to physical and metabolic homeostasis [26]. GHRH is synthesized and stored in the hypothalamus and transported to the pituitary gland where it activates signaling by binding to a specific receptor (GHRH-R) on the pituitary. GH is stimulated by GHRH and secreted by somatotropes in the anterior part of pituitary. Circulating GH exerts its influence by directly binding to a range of cell types with GH receptors or by indirect interaction with IGF-1 [25]. IGF-1 is produced mostly by liver and muscle and regulates cell proliferation, differentiation, and maturation in multiple tissues such as bone, cartilage, skeletal muscle, adipocyte, and cardiomyocyte. By crosstalk with the IGF-1 signaling pathway, GHRH-GH contributes to fundamental physiology, metabolism, and organismal growth including epidermis, connective tissue and bone, wound healing, and blood homeostasis including glucose and lipid control [27]. Circulating GH levels are regulated through long-loop feedback and short-loop feedback mechanisms of GHRH/GH/IGF-1 axis. Because GHRH communicates both through the GHRH/GH/IGF-1 axis and by direct binding to GHRH-R on periphery cells, there is a huge therapeutic potential for its analogues both agonist and antagonist to treat disease that may be associated with any imbalance of GHRH/GH secretion [28].

### 2.2. GHRH Agonists

GHRH agonists are analogues of native human GHRH that have chemically modified amino acid sequences. They were initially synthesized as substitutes to treat growth hormone deficiency (GHD) [29, 30]. Since the agonists exhibit higher activity and better stability compared with the natural GHRH, they are even more suitable for clinical application [43].

In addition to their well-documented ability to stimulate the GH secretion, GHRH agonists affect peripheral tissues by direct receptor binding and stimulating cell proliferation. Multiple cell types may be affected; for example, Dioufa et al. reported that the GHRH agonist JI-38 enhanced wound healing by activating fibroblasts via a fibroblast splice variant of the native GHRH receptor [31, 32]. The GHRH agonist MR-403 was shown to have a cytoprotective effect on rat islet cocultured with adrenal cells [44]. Recently a series of new selective and more potent GHRH agonistic analogues have been produced and are being developed for clinical application. These include MR-356, MR-361, and MR-367 [45].

For cardiovascular indications, GHRH-R was recently found to be expressed by rat cardiomyocytes and administration of exogenous GHRH blocked apoptosis and reduced the cardiac scar size after myocardial infarction. The effects correlated with GHRH-R-mediated activation of reperfusion injury salvage kinase (RISK) and other survival kinase pathways including extracellular regulated protein kinases (ERK) 1/2, phosphatidylinositol 3-kinase (PI3K)/Akt and adenylate cyclase (AC)/cyclic adenosine monophosphate (cAMP)/protein kinase A (PKA), glycogen synthase kinase-3*β*, and the signal transducer and activator of transcription-3 (STAT3) signaling pathways [33, 34]. In an extension of this, subcutaneous administration of the potent GHRH agonist MR-409 was recently shown to exert powerful cardioprotection in a swine AMI model [35]. Application of MR-409 did not affect the heart weight/body weight index and was without any detectable adverse effects supporting safety in a large animal model. The authors concluded that systemic GHRH agonists protect the heart and preserve cardiac function during and after infarction by activating GHRH receptors on cardiomyocytes. Other studies confirm the cardioprotective, reparative functions of GHRH agonist administration and have demonstrated significant downregulation of inflammatory cytokines including IL-2, IL-6, and IL-10 in response to such agonists in vivo [36]. GHRH agonist JI-38 was shown to increase SDF-1 expression and stem cells homing in a rat model of AMI, thereby promoting cardiac repair and angiogenesis [2]. Therefore, GHRH and its agonists may play important roles in the integrity and resilience of peripheral tissues, including the heart and coronary vasculature.

### 2.3. Antitumor Effects of GHRH Antagonists

GHRH and GHRH-R are expressed in diverse tumor cells including human breast, endometrial, and ovarian cancers. Such locally generated GHRH circuits in tumor cells trigger bioactive and immune responses that directly impact tumor cell proliferation and expansion. Plasma GHRH levels under normal (nontumorigenic) conditions are low to undetectable because of rapid clearance and there is sparse evidence that naturally circulating GHRH contributes to tumorigenesis [46]. However, GHRH antagonists may be able to suppress the tumor progression by counteracting the localized circuits of GHRH/GHRH-R that are active in the tumor microenvironment. GHRH antagonists act as competitors of GHRH for binding to GHRH-R thereby blocking GHRH-R activation [47]. This provides us a novel approach to treat cancer with GHRH antagonists. For the past 25 years the antitumor properties of GHRH antagonists have been studied on cancer cell lines from breast, prostate, pancreas, colon, lung, ovarian, brain, and lymphocyte [23, 24, 37–42]. GHRH was shown to cause MAPK activation in MDA-MB-231 breast cancer cells via phosphorylation of Ras and Raf, and the GHRH antagonists MZ-J-7-138 and JV-1-92 were shown to block this pathway and suppress lung carcinoma growth in a manner that correlated with Ras inhibition [40, 48]. It was further demonstrated that the GHRH antagonist JMR-132 inhibited prostate cancer cell growth by suppressing Akt and ERK pathways [49]. All these studies confirm the complexity of growth regulation and signaling pathways in tumor cells that expose multiple potential targets for GHRH and its antagonists.

**Figure 1:**
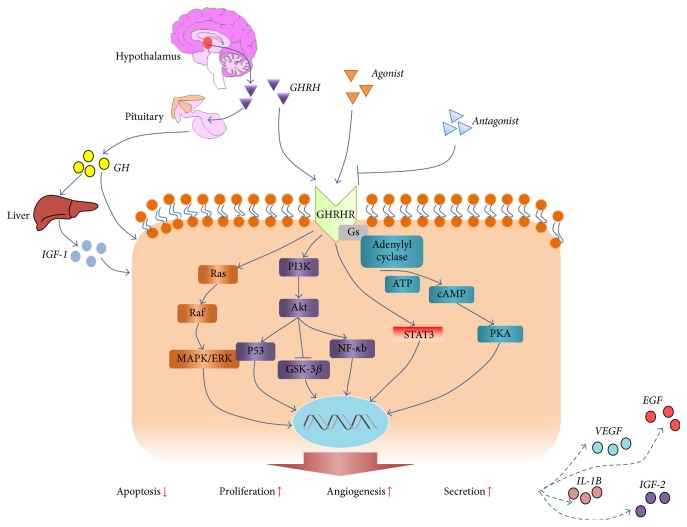
Cellular effects of GHRH analogues. GHRH is secreted by the hypothalamus and binds to GHRH-Rs on the pituitary to stimulate secretion of GH and downstream activity of IGF-1. GHRH and its agonists can bind directly to GHRH-Rs on multiple cell types of endocrine and nonendocrine origin. Signaling pathways that are activated by GHRH and its agonists include AC/cAMP/PKA, Ras/Raf/ERK, PI3K/Akt, and STAT3. Mediation through these signaling pathways leads to enhanced cell survival, proliferation, and secretion of cytokines. GHRH antagonists inhibit these pathways by competitively binding to the GHRH-R.

Angiogenesis is a central activity that controls the growth, expansion, and metastasis of tumors [50], which has been used as a primary target for the antitumor actions of GHRH antagonists. The GHRH antagonist MZ-J-7-114 was shown to block the activities of VEGF and downregulate the expression of epidermal growth factor (EGF) and VEGF receptors, thereby effectively abolishing angiogenesis and tumor growth [5]. The effects of GHRH analogues and their discriminative roles on normal versus tumor cells are summarized in Figure 1 and Table 1.

### 2.4. Interaction between GHRH and Other Hormones

GHRH regulation of GH secretion through GHRH/GH/IGF-1 is well established. GH secretion is also regulated indirectly by GHRH interactions with other hormones. Ghrelin is a 28-amino acid peptide produced by cells in the gastrointestinal tract that regulates GH release in a dose dependent manner [51]. Ghrelin is a more potent stimulator of GH release than GHRH and the combined effect of ghrelin and GHRH on GH release is additive. GHRH and ghrelin bind independently to GHS and GHRH receptors with corresponding effects downstream [52]. Ghrelin activates the hypothalamic-pituitary-adrenal axes to regulate sleep. GHRH stimulates slow-wave sleep while corticotrophin-releasing hormone (CRH) antagonizes these pathways and stimulates wakefulness. CRH can strengthen the ghrelin-induced cortisol secretion but has no direct effect on GH release, while GHRH opposes CRH [53]. Interactions between GHRH, ghrelin, and sex steroids are synergistic and short-term beta estradiol application in postmenopausal women enhances ghrelin efficiency in the presence of GHRH [54].

## 3. MSCs Promote Angiogenesis

MSCs are multipotent stem cells that can differentiate into multiple cell lineages including osteoblasts, chondrocytes, adipocytes, myoblasts, fibroblasts, and stromal cells. MSCs can also be stimulated to express markers of cardiomyocytes, hepatocytes, and endothelial cells [55]. Based on their broad regenerative and immune-privileged properties, MSCs have been widely tested for use in regenerative medicine, in particular myocardial infarction and the related promotion of angiogenesis and vasculogenesis [56–64]. MSCs promote angiogenesis by the following actions: (1) secretion of trophic factors such as VEGF-A and chemoattractive cytokines [65]; (2) organization of pericytes and endothelial support cells during neovascularization [66, 67]; (3) stimulation of endogenous endothelial regenerative progenitor cells (EPCs) during ischemic injury [68]; (4) immune regulation of the microenvironment to enhance cell survival and angiogenesis [69, 70].

### 3.1. Paracrine Effects of MSCs

The proangiogenic stimuli of MSCs are widely believed to be mediated by paracrine pathways particularly through the actions of VEGF-A, *β*FGF, and SDF-1*α* [65, 71, 72]. Cysteine-rich protein 61 (Cyr61 or aka CCN1) is a novel proangiogenic factor that belongs to the CCN family. Cyr61 is secreted by MSCs and contributes importantly to the paracrine proangiogenic effect especially in damaged tissues [10]. Capillary growth requires degradation of the surrounding extracellular matrix (ECM) to allow endothelial cell sprouting [66]. Matrix metalloproteinases including MMP2, MMP9, and MMP14 are secreted by MSCs and target the ECM through distinct proteolytic actions thereby modulating the microenvironment and promoting appropriate interactions between MSCs and endothelial cells [66]. GATA-4 is a zinc finger transcription family that plays a key role in regulating the proangiogenic paracrine actions of MSCs. Overexpression of GATA-4 in MSCs enhances the angiogenic actions of MSCs by augmenting the secretion of multiple proangiogenic factors including VEGF-A, IGF-1, and vWF [73]. MSCs stimulate upregulation of VE-cadherin and recruitment of *β*-catenin to endothelial cells, which are vital for the integrity of endothelial barriers and junctions [74]. MSCs also secrete a rich mixture of cytokines and immune-modulating factors that enhance angiogenesis directly and indirectly.

### 3.2. MSC Function as Pericytes and Vascular Cells

MSCs can function as perivascular cells or pericytes that accumulate around the vessels in the MSC niche [8]. These cells provide structural support and may constitute a reservoir of undifferentiated precursor cells for tissue regeneration [75]. Pericytes and MSCs share common cell surface markers and are both multipotent [76]. Pericytes may be viewed as vascular MSCs that can migrate under appropriate stimulation from the MSC vascular niche to the vascular tube where they regulate the neovascularization by secreting bioactive cytokines such as VEGF-A [77]. The high migratory capability of MSCs allows them to be recruited to multiple targets in vivo, including damaged tissues and tumors such as glioma where they can integrate as pericytes. As such MSCs may be used as selective antitumor drug transfer vehicles [78].

It has also been shown that the pericytes or vascular MSCs present in the aortic artery may modulate restenosis after arterial injury. Engrafted MSCs with endothelial-like phenotypes were shown to express endothelial nitric oxide synthase (eNOS) and may retard the processes of restenosis [79]. In contrast to this, other research suggests that MSCs preferentially migrate to the medial zone of blood vessels and differentiate into vascular smooth muscle cells [80]. Therefore, the direction of MSC differentiation may be related to the tissue specific microenvironment. When cultured under endothelial differentiation conditions, MSCs express endothelial traits and markers possibly through the upregulation of forkhead box protein C2 (FOXC2) and its downstream target alpha v beta 3 integrin/CD6 [81]. VEGF-A stimulation of cultured MSCs also promotes endothelial-like differentiation by activating the Rho/Rho-associated coiled-coil containing protein kinase (ROCK) signaling pathway and myocardin-related transcription factor-A (MRTF-A) [82]. Physical stimuli such as modeled microgravity with or without VEGF can direct MSCs to express endothelial markers including Flk-1 and vWF [83, 84]. Taken together these results confirm the multipotency of MSCs and support major roles in vascular regeneration including possible direct contributions to endothelial cell recruitment and sprout formation.

### 3.3. Stimulation of Endogenous Regenerative Programs

EPCs may contribute to the structure, organization, and architecture of regenerating blood vessels. In addition to their potential to generate mature endothelial cells, EPCs also mediate paracrine actions by secreting angiogenic cytokines especially at the early stages of vessel formation [85]. EPCs and MSCs cross-communicate at multiple levels and when cocultured both types of cells demonstrated enhanced proliferation and proangiogenic properties [86]. MSC-EPC intercellular signaling involves both paracrine effects and direct cell-to-cell communication [87, 88]. Simultaneous tissue transplantation of MSCs with EPCs promotes angiogenesis in a synergistic manner and conditioned medium from cocultures of EPCs and MSCs generate complementary sets of angiogenic cytokines and more efficiently promote angiogenesis of cultured endothelial cells under both normoxic and hypoxic culture conditions [89]. The results support synergism between EPCs and MSCs at multiple levels in the developing, regenerating vasculature, particularly involving secreted cytokines that promote cell recruitment, proliferation, and differentiation [90].

### 3.4. Immune Regulation

MSCs modulate immunoreactions by interacting with immune cells. MSCs inhibit the proliferation and maturation of B-cells and NK-cells and suppress the proliferation of CD4^+^ and CD8^+^ T-cells while wielding protective effect on other cells such as neutrophils [91]. MSCs can also inhibit lymphocyte proliferation as well as B-cells differentiation [55]. However, the precise mechanisms of MSCs-related immune regulation properties are not fully understood. A number of distinct pathways have been described. Firstly, MSCs can modulate immune cells by secreting related chemokines and cytokines. Activation of Toll-like receptors 3 and 4 on MSCs increases the production of IL-6, IL-8, and chemokine (C-X-C motif) ligand 10 (CXCL10) to suppress the proliferation of T-cells through Notch signaling [92]. Secondly, MSCs attract proinflammatory M1 macrophages through secreted cytokines CXCL2, macrophage inflammatory protein-1*α* (MIP-1*α*), MIP-1*β*, and growth-regulated protein *β* [69, 93]. Thirdly, MSCs can recruit anti-inflammatory M2 macrophage by accumulated production and secretion of PEG2 under mediation by *γ*-IFN to balance the inflammatory responses [94]. Furthermore, MSCs can directly contact to endothelial cells by the functional adherens junctions mediated by VE-cadherins and *β*-catenin to maintain intact endothelial barriers. Endothelial barriers can prevent uncontrolled inflammation that would be caused by increased vascular permeability [74]. Immune regulation by MSCs helps support an appropriate microenvironment that is conducive to angiogenesis and the maturation of new blood vessels.

### 3.5. Preconditioning to Enhance the Viability of MSCs

Despite their unique properties, clinical applications of MSCs for tissue protection and regeneration are limited by poor survival and limited engraftment. Multiple preconditioning stimuli have been tested to improve posttransplantation survival and function. These include gene modification, physical and chemical preconditioning, and the pretransplantation culture microenvironment.

Physical preconditioning includes hypoxia preconditioning [95, 96], which can attenuate apoptosis caused by hypoxia/reoxygenation. Our group has shown that the superior performance of MSCs conferred by hypoxia precondition is mediated at least in part through a leptin-mediated mechanism [15]. Hydrogen gas and oxidative stress pretreatments were also successful preconditioning stimuli that improved survival of pretransplantation of MSCs [97, 98].

Paracrine stimulation by multiple growth factors, cytokines, and chemicals can confer protection and increased survival. The repertoire includes growth factors such as TGF-*β* [99], TGF-*α* [99], erythropoietin (EPO) [17], epidermal growth factor [100], parathyroid hormone [101], and K-channel activator diazoxide [102, 103]. Such preconditioning can be implemented by pretreatment with recombinant proteins or by gene modification. For example, MSCs that overexpress GSK-3*β* by gene transfection have significantly improved cardioprotective properties compared with MSCs alone [104]. Similarly, MSCs that overexpress the surviving gene conferred superior recovery in a rat myocardial infarction model [105]. In a rapidly advancing research field of biomaterial engineering, it has been shown that 3-dimensional support systems that mimic the microenvironment of the ECM support markedly improved cell survival after transplantation. Such engineered scaffolds are being tested to deliver sheets of supportive cells to the myocardium and islets for treating diabetes and bone reconstruction [106, 107].

**Figure 2:**
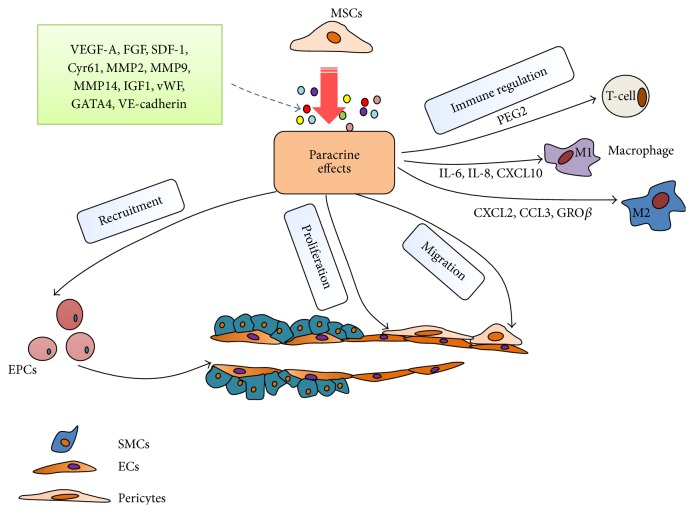
Proangiogenic roles of MSCs. MSCs promote angiogenesis by (1) secreting proangiogenic bioactive factors; (2) functioning as pericytes to support EC proliferation and maturation; (3) recruiting EPCs and other progenitor cells into neoblood vessels; (4) regulating the immune response of the microenvironment by modulating recruitment of immune cells.

In summary multiple pretreatment strategies have been developed to enhance the performance of MSCs as ex vivo reagents for angiogenesis and tissue repair. To our knowledge none have been tested clinically but preclinical results indicate efficacy and safety of most protocols and support clinical translation. The known roles of MSCs in promoting angiogenesis are summarized in Figure 2.

## 4. Functional Enhancement of MSCs by GHRH Agonists 

### 4.1. Expression of GHRH Receptor on MSCs

In addition to the hypothalamus and pituitary, GHRH receptors are expressed in multiple extrahypothalamic tissues including renal medulla, renal pelvis, heart, lung, small intestine, and testis [108, 109]. Our laboratory and others have shown that GHRH receptors are also expressed on MSCs [20, 110].

### 4.2. GHRH Analogue Exerts Similar Effect as Proangiogenesis Ability of MSCs

GHRH and its analogues promote cell proliferation in multiple target tissues by binding and activating the GHRH receptor. GHRH analogues may have improved therapeutic properties compared with the parent GHRH. GHRH analogues have been shown to augment VEGF-A secretion in the contexts of neuroendocrine tumor cells and myocardial infarction [1, 2]. It was recently shown that treatment of MSCs from S-nitrosoglutathione reductase (GSNOR) knockout mice with a GHRH analogue JI-38 enhanced VEGF production and stimulated the angiogenic potential of JI-38-treated MSCs [20]. JI-38 appears to stimulate a paracrine circuit by binding to GHRH-R on MSCs promoting VEGF secretion and proangiogenesis.

### 4.3. Regulation of MSC Proliferation by GHRH Agonists

In one of its classical pathways, GHRH stimulates cell cycle entry and proliferation via activating adenylate cyclase and subsequently stimulating PKA to increase influx of Ca^2+^ through plasma membranes [111, 112]. GHRH stimulation also activates phospholipase C (PLC), increasing the production of inositol phosphates (IP) and diacylglycerol (DAG) and activating PKC in pituitary somatotrophs [113]. The same signaling pathways may cause enhanced proliferation by GHRH agonists and other factors when GHRH-Rs are activated in MSCs. Previous studies confirmed classic signal activation by cAMP and PKA in MSCs. In addition, a new factor, Epac1, was discovered as an exchange protein activated by cAMP, which leads to the activation of Ras-related protein 1 (Rap1) to further trigger the Akt phosphorylation. Activated (p)-Akt is a central regulator of insulin and IGF-1 signaling and can promote translocation of *β*-catenin to nucleus to augment the expression of c-myc and VEGF thereby controlling MSC proliferation [114].

As discussed earlier in this review, GHRH and its agonists promote the proliferation of pituitary cells through the activation of ERK1/2 and JAK/STAT3 signaling pathways [115]. These pathways are also required for GHRH to activate MSC proliferation. Indeed, the canonical mitogen-activated protein kinase (MAPK) signaling systems are well known to exert major control over cell metabolism, growth, differentiation, and cell death/survival pathways. Jaiswal et al. recently reported that MAPK is centrally involved in the process of proliferation and differentiation of human MSCs into osteogenic lineage [116]. GHRH agonists and analogues are likely to use to the same pathways to stimulate MSC proliferation.

### 4.4. Antiproliferative Role of GHRH Antagonists on Neoplasms

GHRH and its receptor have been found in many tumor cell lines and neoplasms [111, 117–119]. GHRH can stimulate tumor growth through the GHRH/GH/IGF-1 axis and local GHRH stimulatory loops between GHRH and its receptor. By obstructing the interaction of GHRH with its receptor using an GHRH antagonist, it should be possible to prevent or reduce tumor cell hyperplasia by this pathway [1]. Molecules known to play important roles in cell proliferation including cAMP, PKC, and p21 have been shown to be the effective inhibited targets of GHRH antagonists for their antiproliferation and tumor suppressive actions [37, 120, 121]. These signaling intermediates also regulate MSCs proliferation.

### 4.5. Direct Effect of GHRH Agonists on MSCs

**Figure 3:**
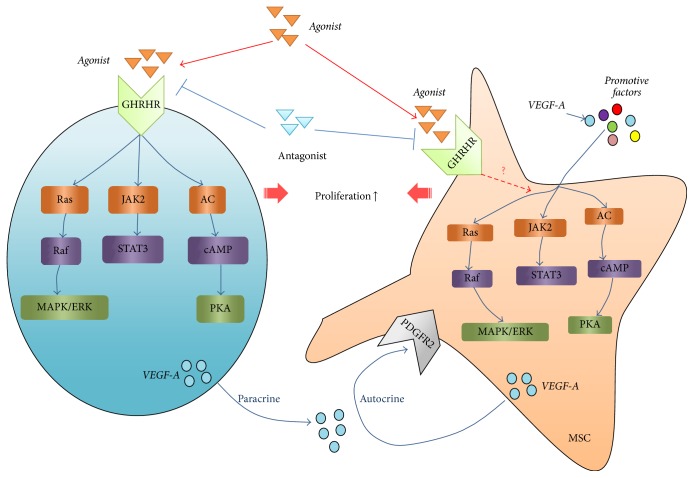
Effects of GHRH agonists on MSCs. Multiple cell types including MSCs express the GHRH receptor and can respond to GHRH agonists and antagonists. Receptor activation communicates with diverse survival pathways that transmit paracrine and autocrine signals to promote cytoprotection, antiapoptosis, and angiogenesis.

Treatment of MSCs with GHRH agonist JI-34 was shown to significantly increase VEGF expression in human MSCs [20]. Recently we reported that pretreatment of MSCs with GHRH agonist JI-38 stimulated cell proliferation, ameliorated apoptosis caused by hypoxia and serum deprivation, and enhanced the proangiogenesis conferred when pretreated cells were injected into ischemic hindlimbs of mice [110]. This is the first report to implicate direct interactions between an GHRH agonist and MSCs. In another report, pretreatment of ckit+ cardiac stem cells with JI-38 also conferred significant cytoprotection that was attributed to activation of ERK and Akt survival pathway [122]. Similar pathways may mediate the effects of JI-38 on MSCs described by our group. The avenues whereby GHRH agonists may enhance cytoprotection and angiogenesis are summarized in Figure 3.

## 5. Clinical Prospects 

So far GHRH analogues have shown promise in preclinical studies. For other hypothalamic hormones, many clinical applications already exist. As early as 1971, Schally et al. isolated luteinizing hormone-releasing hormone (LH-RH) and later introduced analogues of LH-RH into clinical use as antitumor drugs [123]. These studies were extended to advanced endocrine therapy for hormone-related diseases. Schally's team also were the first to describe GHRH antagonists and their possible application for cancer treatment [124]. The recent demonstrations by our group and others, respectively, that JI-38 markedly enhanced the functional properties and potential therapy by MSCs and cardiac stem cells in different models of ischemia raise the possibility that GHRH agonists may be useful agents to augment clinical cell therapy. At the present time MSCs or cardiac stem cells are the leading candidates for such therapy. Because multiple approaches have been used for “precondition” it will be important to compare safety and efficacy side by side in the same study to determine the optimal conditions for clinical trials.

## 6. Conclusion 

GHRH and its analogues regulate cell growth, proliferation, differentiation, and survival through complex networks of signaling pathways involving GHRH/GH/IGF-1 and/or local activation of GHRH receptors on extrapituitary systems and paracrine/autocrine loops. Agonists of GHRH can induce a prolonged stimulation of growth and proliferation via the cAMP/PKA and ERK and JAK2/STAT3 signaling pathways, while the antagonists of GHRH block these effects by competitively binding to the receptor. Regulation by GHRH analogues of the proliferation of normal tissues and neoplasms provides for promising approaches to clinical treatments directed at tissue repair and antitumor treatments.

MSCs are leading candidates for tissue repair and angiogenesis. The superiority of MSCs is attributed to their pluripotency, immune-privilege, and rich factories of secreted paracrine factors that enhance cell survival and confer cytoprotection and angiogenesis. GHRH agonists are one of several potential candidates that confer a powerful “preconditioning” effect to MSCs that enhances viability, mobility, and therapeutic potential. Through the activation of GHRH receptors on the surface of MSCs, GHRH agonists exert cytoprotection of MSCs and confer enhanced angiogenesis. The current results warrant further studies to compare different preconditioning strategies and begin the process of translating the most promising to clinical trials.

## Figures and Tables

**Table 1:** Effects of GHRH and its analogues.

	GHRH	GHRH agonists	GHRH antagonists	Differences between GHRH and analogues
Promote GH secretion	++ [25–28]	++ [29, 30]	—	Quantitative
Cell proliferation	/	+ [31, 32]	—	Qualitative and quantitative
Cardiac protection (reduce infarct size, ameliorate cell apoptosis, and restore heart function)	/	+ [33–36]	—	Qualitative and quantitative
Antitumor effect (suppress tumor cell proliferation and angiogenesis)	—	—	+++ [23, 24, 37–42]	Qualitative and quantitative

Note: +, ++, and +++ represent positive effect: + for mild effect, ++ for moderate, and +++ for significant and strong effect; — represents suppressive effect; / represents no effect.
